# Pronostic de la grossesse qui saigne au premier trimestre: à propos de 239 cas colligés au Centre Hospitalo-Universitaire de Kamenge, Bujumbura

**DOI:** 10.11604/pamj.2020.35.111.20413

**Published:** 2020-04-09

**Authors:** Etienne Kajibwami Birindwa, Jean-Baptiste Sindayirwanya, Salvatore Harerimana

**Affiliations:** 1 Université Catholique de Bukavu, Bugabo 02, Avenue de la Mission, Commune de Kadutu Bukavu, République Démocratique du Congo; 2 Université du Burundi, Avenue de l'Unesco numéro 2, BP 1550 Bujumbura, Burundi

**Keywords:** Métrorragie, pronostic, premier trimestre, Metrorrhagia, prognosis, first trimester

## Abstract

L’objectif de cette étude était d’évaluer le pronostic de la grossesse qui saigne au premier trimestre à l'issue d'un épisode d'hospitalisation d'une femme en urgence à la maternité du Centre Hospitalo-Universitaire de Kamenge (CHUK) en fonction de l'âge de la patiente, de la quantité des saignements ainsi qu'en fonction des constats échographiques. Étude rétrospective descriptive et analytique menée sur 239 dossiers des patientes ayant été hospitalisées sur une période de six ans; de janvier 2012 à décembre 2017. Dans cette étude, le pronostic des grossesses avec métrorragies du premier trimestre au CHUK est mauvais; la majorité a abouti à une fausse couche précoce avec un taux de 65,7%. L'abondance des saignements, l'âge maternel inférieur à 20 ans ou bien supérieur ou égal à 35 ans, sont des facteurs de risque importants mais bien plus la découverte échographique d'un décollement trophoblastique. Il est intéressant de réaliser une étude prospective afin de déceler les étiologies de ces métrorragies et déterminer les complications tardives sur les grossesses qui se poursuivent.

## Abstract (en)

The purpose of this study is to evaluate the post-discharge prognosis of pregnant women with vaginal bleeding during the first trimester, admitted to the Emergency Maternity at the Kamenge University Hospital (CHUK), according to patients age, amount of bleeding and ultrasound results. We conducted a retrospective descriptive and analytical study of 239 medical records of patients hospitalized over a period of six years from January 2012 to December 2017. In this study, the prognosis of pregnant women with first-trimester metrorrhagia hospitalized at the CHUK was bad; the majority of them (65.7%) had miscarriage. Amount of bleeding, maternal age below 20 years, or well above or equal to 35 years, were significant risk factors but much more ultrasound detection of trophoblastic detachment. It would be interesting to perform a prospective study to detect the causes of these metrorrhagias and to determine late pregnancy complications.

## Introduction

Les métrorragies du premier trimestre constituent le motif de consultation le plus fréquent en début de grossesse. Elles suscitent une incertitude quant à l'évolution de celle-ci. Dans les publications occidentales, la fréquence des métrorragies du premier trimestre varie entre 11 et 35% [1-3]; 50% de ces grossesses évoluent jusqu'à terme [4]. En Afrique subsaharienne, la mortalité maternelle, du reste encore très élevée, est due en grande partie aux hémorragies en période périnatale. Les métrorragies du premier trimestre peuvent constituer un prodrome chez les patientes susceptibles de présenter des complications dans l'évolution de leurs grossesses, notamment les hémorragies de la délivrance [5-10]. Cependant, malgré ces connaissances, nous ne retrouvons pas des publications africaines évaluant l'évolution des grossesses ayant un antécédent de métrorragies au premier trimestre. Nous nous proposons ainsi d'étudier rétrospectivement le pronostic de la grossesse qui saigne au premier trimestre à l'issue d'une hospitalisation en urgence à la maternité du Centre Hospitalo-Universitaire de Kamenge (CHUK), dans un premier temps, dans la perspective d'une étude prospective plus large qui permettra d'en déterminer les étiologies et les complications les plus fréquentes survenant sur les grossesses qui se poursuivent, en particulier l'hémorragie de la délivrance.

## Méthodes

Nous avons effectué une étude rétrospective descriptive et analytique sur une période de six ans; du 1^er^ janvier 2012 au 31 décembre 2017 à la maternité du CHUK à Bujumbura, une structure de niveau tertiaire au Burundi. Nous avons inclus dans l'étude toute patiente enceinte de 14 semaines d'aménorrhée (SA) au plus ayant été hospitalisée en urgence à la maternité durant la période d'étude pour des métrorragies et dont la grossesse était évolutive au moment de la consultation. L'âge retenu de la grossesse était calculé à partir de la date des dernières règles ou reconstituée à partir d'une échographie quand celle-ci était disponible.

Nous nous sommes intéressés principalement aux caractéristiques suivantes: la quantité de saignement; légère, modéré, sévère. Nous sommes restés fidèles à la quantification telle que cela été repris dans le dossier de la patiente. Dans notre service, la quantification des saignements est faite par comparaison aux règles: saignement léger: quantité inférieure aux règles normales; saignement modéré: quantité comparable aux règles normales; saignement sévère: quantité supérieure à celle des règles normales; la durée des saignements en jours; l'issue de la grossesse à la fin de l'épisode d'hospitalisation. L'étude portait uniquement sur l'épisode d'hospitalisation; de l'entrée pour métrorragies à la sortie, quelle qu'en soit l'issue.

Nous avions élaboré une fiche de recueil des données reprenant les éléments suivants: l'état-civil, l'âge, la résidence, la profession, les antécédents (médicaux, chirurgicaux, obstétricaux et gynécologiques), les données de la clinique et les traitements. Les données étaient recueillies à partir des registres d'hospitalisation, des registres des mouvements des patientes ainsi que les dossiers d'hospitalisation. Elles étaient saisies dans le logiciel Microsoft Excel 2010 et analysées à l'aide du logiciel Epi info 7. L'analyse des variables était faite par le calcul du Chi-carré avec un degré de signification p ≤ 0,05. Nous n'avions pas inclus dans cette étude les patientes qui présentaient une grossesse môlaire, une grossesse extra-utérine, une interruption volontaire de grossesse (IVG), une fausse couche complète (FCC) ou incomplète (FCI); les patientes ayant un antécédent connu de coagulopathie ainsi que les dossiers d'hospitalisation incomplets; les patientes ayant été vues uniquement en consultation externe non plus n'étaient pas retenues.

## Résultats

**Figure 1:**
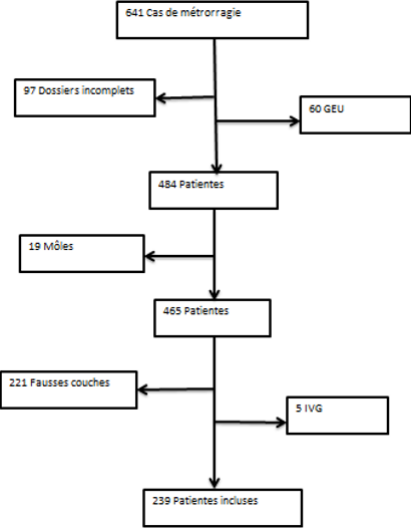
Diagramme de flux des patientes

Sur les six années d'étude, nous avions répertorié 22 674 patientes admises en urgence à la maternité du CHUK parmi lesquelles 21 634 accouchements, 641 cas de consultation pour métrorragies du premier trimestre et 399 cas de consultation pour des motifs divers. Deux cent trente-neuf patientes sur 22 674 remplissaient les critères d'inclusion; soit une fréquence de 2,96% par rapport aux accouchements totaux (Figure 1). La moyenne d'âge des patientes était de 29,53 ans avec des extrêmes de 14 et 49 ans. Les patientes âgées entre 26 et 35 ans représentaient un peu plus de la moitié de l'échantillon (58,16%). La moyenne d'âge des grossesses était de 10,42 SA avec des extrêmes de 5 et 13,43 SA. En fréquence, la tranche de 9-10 SA était la plus représentée soit 33,89% des cas (Tableau 1). Plus de la moitié des patientes (n = 152-63,60%) présentaient un saignement léger suivi de celles ayant eu un saignement modéré (22,59%). Les métrorragies avaient duré un jour dans 69,87% des cas.

**Tableau 1:** Caractéristiques des patientes

Caractéristiques	Effectif	Pourcentage (%)
**Tranche d’âge (ans)**		
≤ 20	54	22,59
21-25	10	4,18
26-30	71	29,71
31-35	68	28,45
> 35	36	15,06
**Total**	**239**	**100**
**Age de la grossesse (semaines d’aménorrhée)**		
< 7	8	3,35
7-8	49	20,50
9-10	81	33,89
11-12	67	28,03
13-14	34	14,23
**Total**	**239**	**100**

Sur le plan échographique, 76 patientes (31,8%) avaient une grossesse évolutive sans signe de décollement trophoblastique; 74 autres (30,96%) avaient une grossesse évolutive avec décollement trophoblastique alors que 37,24% étaient porteuses d'une grossesse d'évolutivité incertaine (Tableau 2). Seules quatre-vingt-deux patientes (34,31%) étaient porteuses d'une grossesse évolutive à la sortie; 65,69% avaient eu une fausse couche en cours d'hospitalisation, parmi lesquelles 41,42% avaient un saignement léger et 10,04% avaient eu un saignement sévère; dans ce dernier groupe, plus de la moitié a avorté; environ 73%. Les femmes âgées de 20 ans ou moins et celles âgées de plus de 35 ans ont le plus perdu leur grossesse; respectivement dans l'ordre de 70 et 72% néanmoins, l'écart statistique entre les différentes tranches d'âges n'est pas important (Tableau 3). Les grossesses présentant un décollement trophoblastique à l'échographie ainsi que celles d'évolutivité incertaine, évoluaient plus vers une fausse couche avec une différence statistique énorme (Chi-carré = 165,8: p < 0,0001) (Tableau 4).

**Tableau 2:** Répartition des patientes selon la clinique et la para clinique

Caractéristiques	Effectif	Pourcentage (%)
Quantité des saignements		
Légère	152	63,60
Modérée	54	22,59
Sévère	33	13,81
**Total**	**239**	**100**
Résultats d’échographie N=239		
Grossesse évolutive sans décollement trophoblastique	76	31,8
Grossesse évolutive avec décollement trophoblastique	74	30,96
Sac sans embryon (évolutivité incertaine)	89	37,24

**Tableau 3:** Évolution et pronostic par rapport à l’abondance, au terme de la grossesse et à l’âge de la patiente

Variable	Diagnostic de sortie				Total	%	p value
Abondance des saignements	Fausse couche		Grossesse évolutive				
	**Effectif**	**%**	**Effectif**	**%**	**Effectif**		0,6299
Légère	99	65,13	53	34,87	152	100	
Modérée	34	62,96	20	37,04	54	100	
Sévère	24	72,73	9	27,27	33	100	
**Total**	157	65,69	82	34,31	239	100	
**Terme de la grossesse (SA)**							
< 7	4	50,00	4	50,00	8	100	0,7322
7-8	30	61,22	19	38,78	49	100	
9-10	53	65,43	28	34,57	81	100	
11-12	47	70,15	20	29,85	67	100	
13-14	23	67,65	11	32,35	34	100	
**Total**	157	65,69	82	34,31	239	100	
**Tranche d’âge des patientes (années)**							
≤ 20	7	70	3	30	10	100	0,5761
21-25	37	68,52	17	31,48	54	100	
26-30	41	57,75	30	42,25	71	100	
31-35	46	67,65	22	32,35	68	100	
>35	26	72,22	10	27,78	36	100	
**Total**	157	65,69	82	34,31	239	100	

**Tableau 4:** Évolution de la grossesse par rapport à l’échographie

	Diagnostic de sortie					
Résultats de l’échographie	Fausse couche		Grossesse évolutive		Total	
Activité cardiaque présente sans décollement trophoblastique	6	7,9%	70	92,1%	76	100%
Activité cardiaque présente avec décollement trophoblastique	71	95,9%	3	4,1%	74	100%
Evolutivité incertaine	80	89,9%	9	10,1%	89	100%
**Total**	157	65,7%	82	34,3%	239	100%

*P<0,0001

## Discussion

La fréquence des métrorragies du premier trimestre au CHUK était de 2,96% durant la période de cette étude. Celle-est ci est très basse par rapport à la fréquence rapportée dans la littérature qui varie de 11 à 35% [1-4, 11]. Cette différence serait liée à une faible fréquentation des urgences de la maternité du CHUK pour ce problème des métrorragies. Pendant qu'en occident, le moindre saignement vaginal sur grossesse pousse à consulter urgemment le gynécologue, les patientes burundaises consulteraient rarement tant que la situation ne parait pas grave. La pauvreté et le niveau intellectuel bas renforcerait ce comportement, bien que l'Etat offre des soins gratuits aux femmes enceintes. Cette fréquence basse en apparence peut encore être liée à nos critères d'inclusion qui diffèrent de la plupart des études. L'âge des grossesses variait entre 5 et 13,43 semaines; la moyenne d'âge de survenue de saignement était 10,42 semaines.

La tranche de 9 à 10 semaines est plus représentée avec 33,89% des grossesses qui saignent; le terme étant calculé sur base de la date des dernières règles, les grossesses peuvent être jeunes de deux semaines qu'on ne le trouve et ainsi la plupart des saignements surviennent en réalité avant 10 semaines. En effet, il s'agit de la période durant laquelle s'établit la circulation materno-foetale, mais aussi une période d'organogenèse où la grossesse est particulièrement sensible aux « agressions » et donc elles sont plus susceptibles de saigner. Nos constats sont proches de ceux retrouvés dans d'autres études [12-14]. Le risque de saignement avec comme corolaire une fausse couche (FC) présente une relation avec l'âge de la gestante [15]. Yang rapporte un risque relatif (RR) de 1,2 après 35 ans contre 0,7 avant 20 ans [16]. Bien que l'écart statistique du risque de fausse couche entre les tranches d'âges ne soit pas énorme dans notre série (p = 0,5761), nous avons constaté que les femmes âgées de moins 20 ans et celles âgées de plus de 35 ans avaient le plus perdu leur grossesse ; respectivement 70 et 72%. L'âge maternel est associé à une prévalence accrue de fausses couches précoces, notamment après 35 ans; le risque d'aneuploïdie étant augmenté et l'altération la réserve ovarienne ayant commencé [17]. Une étude cas-témoins avec analyse multi variée faite par Maconochie au Royaume-Uni, montre un risque croissant avec l'âge atteignant un odd ratio (OR) de 5,16 à partir de 40 ans [13]. Au Burundi, ce risque serait majoré par les conditions de vie difficiles et la pauvreté; les femmes n'ayant pas d'activité rémunératrice, et donc probablement exposées aux travaux lourds sont plus risque [13].

Dans notre étude, en plus de l'âge des patientes, la quantité de saignement semble influer sur l'évolution de la grossesse ; 73% des femmes ayant saigné de façon importante avaient perdu leurs grossesses. La série d'Hassan [2] a établi une corrélation entre l'abondance des saignements et les antécédents de la femme. Dans une autre série de 4510 femmes [15], cet auteur a retrouvé que le seul fait de saigner n'était pas associé à un risque de FC. Néanmoins, lorsque le saignement était important, il s'observait une forte probabilité de FC avec un OR = 2,97 (95% IC: 1,93; 4,56) surtout quand des douleurs pelviennes coexistaient. L'échographie s'avère une exploration d'appoint pour la confirmation du diagnostic et la prise de décision pour des soins optimaux. Les grossesses présentant un décollement trophoblastique à l'échographie ainsi que celles d'évolutivité incertaine, évoluaient plus vers une fausse couche avec une différence statistique significative (Chi-carré = 165,8: p < 0,0001)). L'existence du décollement trophoblastique/hématome est un élément de très mauvais pronostic. Plusieurs auteurs proposent la définition échographique d'une grossesse évolutive ou non évolutive en fonction des mesures du sac gestationnel (SG), de la longueur cranio-caudale (LCC) de l'embryon, de l'existence ou non d'une activité cardiaque (AC) et des dimensions de la vésicule vitelline (VV). Il en est de même du seuil de vacuité utérine acceptable lors d'une FC [18-20]. Le consensus est que la grossesse est dite arrêtée si on retrouve un embryon de LCC supérieur ou égale à 7 mm sans AC visible ou bien un SG mesurant 25mm ou plus sans embryon. Pour les grossesses n'ayant pas encore atteint ces mensurations, un contrôle deux semaines plus tard est recommandé; il s'agit donc des grossesses d'évolutivité incertaine. L'absence de l'AC après ce délai confirme le diagnostic [20, 21].

Le contenu des comptes rendus échographiques retrouvés dans les dossiers de nos patientes ne nous a pas souvent permis de confirmer si leurs conclusions respectaient ces définitions d'autant plus que la LCC ou le SG n'étaient que rarement mesurés. Seuls 89 comptes rendus rapportaient les mensurations qui nous avaient permis de classer ces grossesses comme étant d'évolutivité incertaine et parmi elles, 80 (89,9%) s'étaient soldées par une FC. L'existence d'un hématome vu à l'échographie chez une gestante au premier trimestre, accroit le risque de FC [22]. Poulose rapporte aussi un risque de FC tardive à 22,6% (p = 0,008) dans ce cas [23]. Le risque de FC précoce augmente lorsque l'activité cardiaque n'est pas visible [14] de même en cas de bradycardie avec une fréquence cardiaque fœtale (FCF) inférieure à 110 battements par minute [24] alors qu'il diminue de 3% lorsqu'une activité cardiaque est vue à l'échographie [25]. La présence d'un hématome constitue un facteur de mauvais pronostic pour l'évolution de la grossesse; ce risque est corrélé au volume de l'hématome [26]. Le manque de la description de l'hématome dans les comptes rendus retrouvés dans les dossiers de nos patientes ne nous a pas permis d'évaluer le risque de FC par rapport au volume de l'hématome ou à l'étendue du décollement. Le manque d'habitude ou bien la précipitation des médecins dans leurs examens devant une patientèle en surnombre sollicitant le CHUK pourraient en être l'explication.

## Conclusion

La fréquence des FC sur les grossesses qui saignent au premier trimestre chez les patientes qui consultent au CHUK est très importante (65,7%) alors que seulement 2,96% des femmes viennent y consulter pour cette plainte. L'âge de la femme inférieur à 20 ans ou bien supérieure à 35 ans, le saignement quelle que soit son abondance ainsi que la découverte échographique d'un décollement trophoblastique en sont des facteurs de risque importants. Les facteurs qui concourent à l'exacerbation de cette survenue des fausses couches par rapport à la littérature sont à élucider (causes infectieuses, travaux lourds, causes génétiques). L'étude étiologique de la survenue de ces fausses couches n'ayant pas été l'objectif de notre étude, nous estimons très intéressant de réaliser une étude prospective plus vaste sur les grossesses saignant au premier trimestre afin d'en étudier l'évolution jusqu'à terme, identifier les complications fréquentes y afférant surtout l'hémorragie de la délivrance et dégager la part des différentes étiologies des FC dans notre milieu; particulièrement les étiologies génétiques, celles-ci étant mises en cause le plus souvent dans les milieux où cette question a déjà été étudiée.

### Etat des connaissances actuelles sur le sujet

- Environ un quart des grossesses saignent au premier trimestre;
- L'abondance des saignements et l'âge maternel inférieur à 20 ou bien supérieur à 35 ans sont des facteurs de risque importants;
- Au moins la moitié évolue jusqu'à terme.

### Contribution de notre étude à la connaissance

- Cette étude, la première de son genre au Burundi, révèle que les burundaises consultent très peu pour les saignements du premier trimestre;
- Plus de la moitié des femmes qui consultent au CHU Kamenge pour cette plainte perdent leur grossesse précocement (66% environ);
- Nos résultats suscitent la curiosité de déterminer les causes de saignement du premier trimestre qui rendent le pronostic illusoire et pousse à savoir quel est l'issue des grossesses qui dépassent le premier trimestre après avoir saigné.

## Conflits d’intérêts

Les auteurs ne déclarent aucun conflit d'intérêts.
